# Identification of DNA methylation signatures for hepatocellular carcinoma detection and microvascular invasion prediction

**DOI:** 10.1186/s40001-022-00910-w

**Published:** 2022-12-05

**Authors:** Yijie Hao, Qingxia Yang, Qiye He, Huanjing Hu, Zongpeng Weng, Zhixi Su, Shuling Chen, Sui Peng, Ming Kuang, Zhihang Chen, Lixia Xu

**Affiliations:** 1 Center of Hepato-Pancreato-Biliary Surgery, The First Affiliated Hospital, Sun Yat-sen University, No.58 Zhongshan Er Road, Guangzhou, 510080 Guangdong Province China; 2 Institute of Precision Medicine, The First Affiliated Hospital, Sun Yat-sen University, No.58 Zhongshan Er Road, Guangzhou, 510080 Guangdong Province China; 3 Department of Oncology, The First Affiliated Hospital, Sun Yat-sen University, Guangzhou, 510080 Guangdong Province China; 4 Singlera Genomics (Shanghai) Ltd., Shanghai, 201203 China; 5 Department of Biology and Medicine, Zhongshan School of Medicine, Sun Yat-sen University, Guangzhou, 510080 Guangdong Province China; 6 Department of Medical Ultrasonics, The First Affiliated Hospital, Sun Yat-sen University, Guangzhou, 510080 Guangdong Province China; 7 Clinical Trials Unit, The First Affiliated Hospital, Sun Yat-sen University, Guangzhou, 510080 Guangdong Province China

**Keywords:** Hepatocellular carcinoma, Microvascular invasion, DNA methylation, Circulating free DNA

## Abstract

**Background and aim:**

Preoperative evaluation of microvascular invasion (MVI) in patients with hepatocellular carcinoma (HCC) is important for surgical strategy determination. We aimed to develop and establish a preoperative predictive model for MVI status based on DNA methylation markers.

**Methods:**

A total of 35 HCC tissues and the matched peritumoral normal liver tissues as well as 35 corresponding HCC patients’ plasma samples and 24 healthy plasma samples were used for genome-wide methylation sequencing and subsequent methylation haplotype block (MHB) analysis. Predictive models were constructed based on selected MHB markers and 3-cross validation was used.

**Results:**

We grouped 35 HCC patients into 2 categories, including the MVI− group with 17 tissue and plasma samples, and MVI + group with 18 tissue and plasma samples. We identified a tissue DNA methylation signature with an AUC of 98.0% and a circulating free DNA (cfDNA) methylation signature with an AUC of 96.0% for HCC detection. Furthermore, we established a tissue DNA methylation signature for MVI status prediction, and achieved an AUC of 85.9%. Based on the MVI status predicted by the DNA methylation signature, the recurrence-free survival (RFS) and overall survival (OS) were significantly better in the predicted MVI− group than that in the predicted MVI + group.

**Conclusions:**

In this study, we identified a cfDNA methylation signature for HCC detection and a tissue DNA methylation signature for MVI status prediction with high accuracy.

**Supplementary Information:**

The online version contains supplementary material available at 10.1186/s40001-022-00910-w.

## Introduction

Hepatocellular carcinoma (HCC) is the third leading cause of cancer-related mortality worldwide [1]. Approximately 50% of patients recur within 2 years after curative hepatectomy [2]. Microvascular invasion (MVI) is one of the most important risk factors for early postoperative recurrence of HCC [3]. The presence of MVI indicates aggressive behavior of HCC [4], and is closely related to increased risk of recurrence and reduced overall survival (OS) [4, 5].

The preoperative evaluation of MVI status is crucial for decision of surgical strategies. For liver resection in patients with high-risk of MVI, anatomic resection with expanding resection margin should be performed if the remnant liver function is sufficient [6]. For liver transplantation, absence of MVI is required for HCC patients [7]. Besides surgical decision-making, for patients with MVI, additional adjuvant therapies after surgery are preferred. A recent randomized controlled study showed that recurrence-free survival (RFS) could be significantly improved in patients with MVI treated with adjuvant transcatheter arterial chemoembolization (TACE) after resection [8]. Currently, the diagnosis of MVI is determined by postoperative histologic examination. Therefore, an increasing number of studies were motivated to predict MVI status preoperatively, and mainly focused on serum alpha-fetoprotein (AFP) and imaging examination [9–13]. However, no stable and effective predictors have been demonstrated.

DNA methylation is one of the well-known patterns of epigenetic regulation. In general, hypermethylation of tumor suppressor genes is an early event in many tumors [14], suggesting the potential of DNA methylation marker to predict changes associated with tumorigenesis and early metastasis. Previous studies have found that aberrant methylation of some specific genes was associated with HCC initiation and poor prognosis, such as *RASSF1* [15, 16]. Recently, a multi-center study found that a three-CpG-based signature consisting of *SCAND3*, *SGIP1* and *PI3*, can predict early recurrence for early-stage HCC [17]. Currently, no feasible DNA methylation marker has been found for preoperative prediction of MVI status.

Circulating tumor DNA (ctDNA) consists of extracellular nucleic acid fragments released into plasma via tumor necrosis, apoptosis, and active secretion of DNA [18]. Recent studies demonstrated that ctDNA has the potential to innovate screening, diagnosis and subtyping of cancer [19], promoting a blood test, that is, liquid biopsy that enables molecular diagnosis of cancer. Compared with tissue biopsy, liquid biopsy is a noninvasive approach that can represent entire picture of tumor and allow for real-time monitoring of molecular changes in tumor [19, 20]. Recent studies found that ctDNA methylation markers can be applied to screening and diagnosis of HCC with accuracy of more than 90% [21, 22]. It is unclear whether ctDNA methylation marker is available for preoperative prediction of MVI in HCC.

In this study, we applied genome-wide methylation sequencing, and a novel DNA methylation biomarker analysis method, methylation haplotype block analysis, to HCC detection and prediction of MVI status. We found that DNA methylation biomarkers performed well not only in HCC detection, but also in MVI status prediction.

## Materials and methods

### Subjects and samples

A total of 60 liver tissues and 59 plasma samples were analyzed, including 35 HCC tissues and 25 matched peritumoral normal liver tissues as well as 35 corresponding HCC patients’ plasma samples and 24 healthy plasma samples. The characteristic information of healthy controls is presented in Additional file 4: Table S1. For HCC patients in our cohort, inclusion criteria were as follows: (i) curative hepatectomy performed as initial treatment and HCC diagnosed pathologically; (ii) Child–Pugh class A-B; (iii) Eastern Cooperative Oncology Group (ECOG) grades 0–1. Exclusion criteria were as follows: (i) extrahepatic metastasis; (ii) patients who underwent palliative resection; (iii) patients with missing data or loss of follow-up. HCC and peritumoral normal liver tissues were frozen by liquid nitrogen immediately after resection and stored at −80 ℃. HCC patients’ plasma samples were collected before surgery and all the plasma samples were stored at −80 ℃. The clinical data were obtained from the prospectively collected database of HCC in the First Affiliated Hospital, Sun Yat-sen University. Our Institutional Ethic Review Board has approved the current study, following Declaration of Helsinki. Informed consent was obtained from each patient and healthy individual.

### Determination of MVI

MVI was defined as the presence of tumor emboli in a vascular space lined by endothelial cells on microscopy. Two pathologists with more than 10 years of experience in HCC pathology reviewed all the specimen slices independently, without knowing the patient's clinical data. Inconsistency was resolved by consulting with another senior pathologist with 20 years of experience in HCC pathology.

### Circulating free DNA extraction from plasma samples

All blood samples were collected before surgery. Collected blood samples were first centrifuged at 1600 g for 10 min at 4 ℃. Supernatant was then transferred to a new tube and centrifuged at 16000 g for 10 min at 4 ℃. Supernatant was stored at −80 ℃.

Circulating free DNA (cfDNA) was extracted using the QIAamp Circulating Nucleic Acid Kit (Qiagen 55114) in accordance to the manufacturer’s instructions, quantified by Qubit 3.0 (ThermoFisher) and stored at −20 ℃.

### Genome-wide methylation sequencing

Briefly, genomic DNA or cfDNA was digested with restriction enzymes and ligated with a methylated adaptor compatible for Illumina sequencing platforms. Ligation products were converted and purified using the MethylCode^™^ Bisulfite Conversion Kit (ThermoFisher Scientific) and were amplified using PfuTurboCx polymerase (Agilent). Libraries were pooled, size-selected using TBE polyacrylamide gel (Thermo Fisher Scientific) and sequenced on Illumina Hiseq X10 platform for pair-ends (150 cycles).

### Sequencing data analysis

Fastq data are trimmed by adapter and the first 2 bases from each end with trim-galore (https://www.bioinformatics.babraham.ac.uk/projects/trim_galore). After reads trimming, both paired-end reads are merged to a single-end reads. The single reads are mapped to Bismark [23] transformed hg19 genome with Bowtie 1 [24]. The mapped bam files are processed by in house scripts extracting the methylation haplotype information.

### Identification of methylation haplotype block (MHB)

MHBs are defined as two adjacent CpG sites with *r*^2^ higher than 0.5. Methylated haplotype load (MHL) is a measurement of the consecutiveness of methylated cytosines. After getting haplotype information MHBs identified in the last step, the MHL is calculated as weighted ratio of consecutively methylated CpG haplotypes of each length within an MHB:

$${\mathrm{MHL}}=\frac{\sum_{i=1}^{l}{w}_{i}\times P({MH}_{i})}{\sum_{i=1}^{l}{w}_{i}},$$

where l is the length of haplotype (the number of CpGs within an MHB); *W*_i_ stands for weight of each length of haplotype (we select l3 putting higher weights to longer haplotypes); P (MH_i_) stands for the fraction of consecutively methylated haplotype of haplotypes with length i.

In contrast with MHL, the un-methylated haplotype load (UMHL) is a measurement of consecutiveness of un-methylated cytosines. The method is also similar to MHL. In brief, it is the sum of fraction of consecutively un-methylated CpG haplotypes of each length of haplotype within an MHB:

$${\mathrm{UMHL}}=\frac{\sum_{i=1}^{l}{w}_{i}\times P({UMH}_{i})}{\sum_{i=1}^{l}{w}_{i}},$$

where l is the length of haplotype (the number of CpGs within an MHB); *W*_i_ stands for weight of each length of haplotype (we select l3 putting higher weights to longer haplotypes); P (UMH_i_) stands for the fraction of consecutively un-methylated haplotype of haplotypes with length i.

### MHB marker selection

We defined 225,025 MHB regions as previously described [25]. Samples with at least 25,000 detected MHBs are included for downstream analysis. The candidate MHBs are required to be detected in no less than 2/3 of the samples, which leads to 40,676 MHBs. Among them, MHBs with MHL standard deviation no less than 0.02 were selected for marker identification, which lead to 22,849 MHBs. From them a final of 1,022 MHB markers were selected using single-side Wilcoxonrank-sum test (FDR < 0.01).

### Modeling and cross-validation

With the makers identified, we built a Breiman’s random forest (RF) model implemented by R package “randomForest” with ntree = 500 and other default settings. Support vector machine (SVM) model was built with radial kernel implement by R package “kernlab”. Cost was set to 1 by default. RBF kernel parameter sigma is tuned over to get the optimal model. The models were tested with 3-cross validation implemented by R package “caret”.

### Gene Ontology analysis

Gene Ontology (GO) analyses were conducted in GREAT, a web-based tool for GO annotation of regulatory regions [26]. Items with FDR < 0.05 were considered as significantly enriched.

### Methylation markers for HCC detection

To discover methylation marker to differentiate HCC from normal liver tissues, unsupervised clustering was performed based on MHL and UMHL scores to unbiasedly visualize the degree of separation between normal liver tissues and HCC tissues. Principle component analysis was also performed to show the degree of separation of methylation scores between normal liver and HCC tissues. Further, to screen potential MHBs which can be used for HCC diagnosis, Wilcoxon signed-rank test was used to identify candidate MHBs whose methylation scores were significantly different between normal liver tissues and HCC tissues (FDR < 0.05). Using these candidate MHBs and their MHL scores as independent variables, two supervised machine learning algorithms, RF and SVM, were separately employed to train and cross-validate predictive models to classify normal liver tissues and HCC tissues. Further, these candidate MHBs and their MHL scores were used to train a predictive model to differentiate HCC patients and healthy individuals’ plasma samples by RF method, followed by 3-cross validation.

### Methylation markers for MVI prediction

To screen potential MHBs which can be used for MVI status prediction, Wilcoxon rank-sum test was used to identify candidate MHBs whose methylation scores were significantly different between MVI- and MVI + HCC tissues (FDR < 0.05). Using these candidate MHBs and their MHL scores as well as UMHL scores as independent variables, RF method was performed to train and cross-validate predictive models to classify MVI- and MVI + HCC tissues. Then, these identified DNA methylation markers were used to classify MVI- and MVI + HCC plasma samples.

### Statistical analysis

The continuous variables were described by mean ± standard deviation or median and quartile, and the categorical variables were described by frequency and percentage. Independent sample t test or Kruskal–Wallis (KW) nonparametric rank-sum test was used to compare the clinical characteristics between MVI-positive and MVI-negative groups for the continuous variables, while chi-square test or Fisher exact test for categorical variables. The area under receiver operator characteristics curve (AUROC), sensitivity and specificity were used to evaluate the performance of the predictive model. Survival curves were represented by using the Kaplan–Meier method compared with log-rank statistics. All the above statistical analysis were performed by R software. Univariate and multivariate logistic regression models were used to evaluate the associations between MVI and the DNA methylation signature or other clinicopathological variables and to estimate odds ratios (ORs) and 95% confidence intervals; variables with *p* < 0.05 were selected for multivariate analysis (SPSS software, version 26.0). Univariate and multivariate Cox proportional hazards models were used to evaluate the associations between RFS and the DNA methylation signature or other clinicopathological variables and to estimate hazard ratios (HRs) and 95% confidence intervals (R software). A two-sided *P* value was considered statistically significant if less than 0.05.

## Results

### Patient demographic and clinical characteristics

**Fig. 1:**
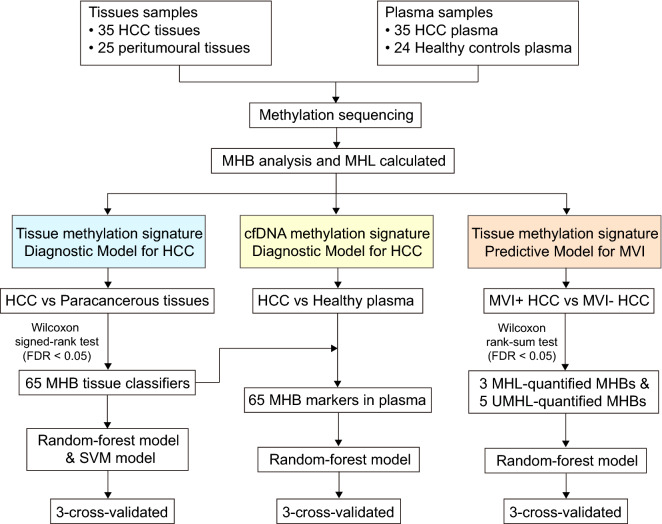
Study flowchart. *HCC* hepatocellular carcinoma, *MHB* methylation haplotype block, *MHL* methylated haplotype load, *MVI* microvascular invasion, *UMHL* un-methylated haplotype load, *SVM* support vector machine

A total of 35 patients and 24 healthy individuals were included (Fig. 1). We grouped 35 patients into 2 categories, including the MVI- group with 17 tissue and plasma samples, and MVI + group with 18 tissue and plasma samples. There were no significant differences in all oncology indicators and liver function indicators between the MVI + group and MVI- group except for AFP, indicating that the baseline data between the two groups were basically balanced (Table 1).

**Table 1:** Baseline demographic and clinical characteristics in MVI− and MVI + groups

Variable	Total (*n* = 35)	MVI− (*n* = 17)	MVI + (*n* = 18)	*P*-value
Age (years)	54.0 (42.0, 62.0)	54.0 (48.0, 64.0)	51.0 (40.5, 61.5)	0.275
Gender				0.554
Male	31 (88.6%)	14 (82.4%)	17 (94.4%)	
Female	4 (11.4%)	3 (17.6%)	1 (5.6%)	
HBsAg				1.000
Negative	3 (8.6%)	1 (5.9%)	2 (11.1%)	
Positive	32 (91.4%)	16 (94.1%)	16 (88.9%)	
AFP (ng/mL)				0.012
≥ 400	10 (28.6%)	16 (94.1%)	9 (50.0%)	
< 400	25 (71.4%)	1 (5.9%)	9 (50.0%)	
Tumor size (cm)	4.0 (3.1, 5.1)	3.5 (3.1, 5.1)	4.0 (3.0, 5.5)	0.974
Tumor number				0.977
Solitary	34 (97.1%)	16 (94.1%)	18 (100.0%)	
Multiple	1 (2.9%)	1 (5.9%)	0 (0.0%)	
Child–Pugh class				1.000
A	33 (94.3%)	16 (94.1%)	17 (94.4%)	
B	2 (5.7%)	1 (5.9%)	1 (5.6%)	
Liver cirrhosis				1.000
Absence	12 (34.3%)	6 (35.3%)	6 (33.3%)	
Presence	23 (65.7%)	11 (64.7%)	12 (66.7%)	
BCLC stage				0.500
0–A	26 (74.3%)	14 (82.4%)	12 (66.7%)	
B–C	9 (25.7%)	3 (17.6%)	6 (33.3%)	
TNM stage				0.369
0–I	30 (85.7%)	16 (94.1%)	14 (77.8%)	
II–III	5 (14.3%)	1 (5.9%)	4 (22.2%)	
Imaging tumor thrombus				0.062
Absence	30 (85.7%)	17 (100.0%)	13 (72.2%)	
Presence	5 (14.3%)	0 (0.0%)	5 (27.8%)	
Tumor necrosis				0.148
Absence	26 (74.3%)	15 (88.2%)	11 (61.1%)	
Presence	9 (25.7%)	2 (11.8%)	7 (38.9%)	
Tumor differentiation				1.000
Well or moderate	30 (85.7%)	15 (88.2%)	15 (83.3%)	
Poor	5 (14.3%)	2 (11.8%)	3 (16.7%)	
Ascites				1.000
Absence	34 (97.1%)	17 (100.0%)	17 (94.4%)	
Presence	1 (2.9%)	0 (0.0%)	1 (5.6%)	
ALB (g/L)	40.2 (37.5, 41.6)	40.4 (38.4, 41.8)	39.5 (36.8, 41.3)	0.530
TBIL (μmol/L)	14.4 (11.4, 21.1)	12.7 (11.3, 19.4)	16.1 (11.8, 22.7)	0.322
ALT (U/L)	33.0 (25.0, 50.0)	33.0 (25.0, 50.0)	33.0 (26.0, 46.5)	0.843
AST (U/L)	33.0 (25.0, 40.5)	34.0 (26.0, 41.0)	32.0 (25.3, 40.0)	0.779
PT (s)	12.1 (11.5, 13.1)	12.1 (11.5, 12.3)	12.1 (11.5, 13.3)	0.667
PLT (10^9^/L)	164.0 (111.0, 206.0)	165.0 (150.0, 200.0)	154.5 (104.25, 203.0)	0.729

Continuous variables are presented as median (inter-quartile range, IQR) unless noted otherwise. Categorical variables are presented as n (%)

*AFP* alpha-fetoprotein, *HBsAg* hepatitis B surface antigen, *ALB* albumin, *TBIL* total bilirubin, *ALT* alanine aminotransferase, *AST* aspartate aminotransferase, *PT* prothrombin time, *PLT* platelet count

### Tissue methylation markers to distinguish HCC tissues from non-tumor tissues

**Fig. 2:**
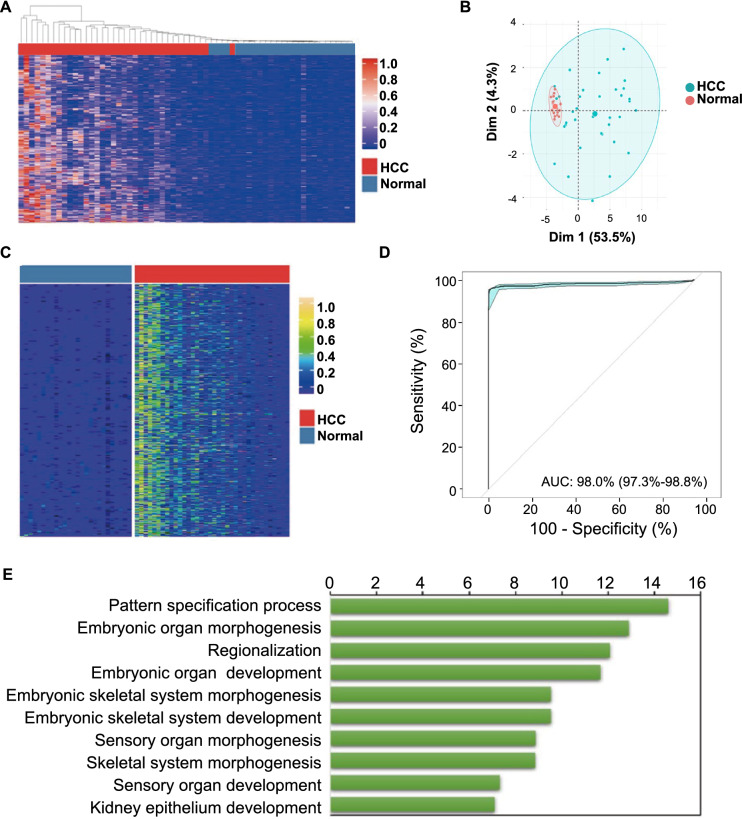
DNA methylation markers classify normal liver and HCC tissues with high degree of accuracy. **A** Unsupervised clustering of normal and HCC tissues based on top 100 MHBs that have the highest degree of variations in their MHL scores; **B** PCA analyses show clear separation between normal liver tissues and HCC tissues; **C** supervised analyses identified 65 MHBs as classifiers for normal liver tissues and HCC tissues; **D** RF-built classification models using the 65 MHB markers accurately classified normal liver tissues and HCC tissues, as was demonstrated by the AUC of their ROC curves; **E** top biological function categories of the identified MHB markers

To screen MHB for classifiers for HCC, we quantified the DNA methylation status of all sequenced MHBs by MHL, and then performed unsupervised clustering based on those scores to visualize the degree of separation between HCC tissues and non-tumor tissues. Results showed that based on MHL scores, the HCC tissues had consistently been separated from the normal liver tissues (Fig. 2A), which demonstrated that there were profound differences in DNA methylation patterns between HCC and peritumoral normal liver tissues. We further performed principal component analyses (PCA) on the MHL scores of normal liver and HCC tissue, and the results showed that the normal liver tissues were also separated from the HCC tissues (Fig. 2B), in agreement with the results observed in unsupervised clustering.

After filtering MHBs and libraries, we performed Wilcoxon signed-rank test to the dynamic MHBs based on their MHL scores, and identified 65 MHBs whose methylation scores were significantly different between normal liver tissues and HCC tissues (False Discovery Rate (FDR) < 0.05) (Fig. 2C). Using these MHBs and their MHL scores as independent variables, and the liver tissues from our study cohort as training and validation sample sets, we separately employed two supervised machine learning algorithms, RF and SVM to train and cross-validate binary predictive models to classify normal or HCC liver tissues. Results showed both RF- and SVM-built models were highly accurate in classifying HCC and normal liver tissues: the AUC is no less than 0.98 (RF model: AUC = 98.0%, 95% confidence interval CI 97.3–98.8%, 10-time repeat, Fig. 2D; SVM model: AUC = 99.9%, 95% CI 99.9–99.9%, 10-time repeat, Additional file 1: Figure S1A).

### Gene Ontology analysis of HCC methylation markers

To investigate the potential biological functions of these methylation markers, especially their roles in the pathology of HCC, genes associated with identified MHBs were annotated and analyzed based on their known biological and molecular functions. Results showed the biological function categories that had the highest level of enrichment were those involved in the general or specific processes of embryonic development/differentiation (Fig. 2E), such as pattern formation, embryonic organ morphogenesis and development, regionalization, sensory organ and skeletal system morphogenesis, etc. This was consistent with the finding that many embryonic genes were re-expressed in cancer cells [27], suggesting that these methylation markers might regulate these embryonic genes’ expression during HCC progression. Indeed, when those genes were re-analyzed based on their molecular function, the top categories with the highest level of enrichment were transcription factors that regulate the expression of other genes (Additional file 1: Figure S1B). This suggests that some of the identified MHB classifiers may be pivotal to the progression of HCC by regulating cascades of gene expression that are signatory to HCC.

### cfDNA methylation signature to distinguish HCC patients from healthy individuals

**Fig. 3:**
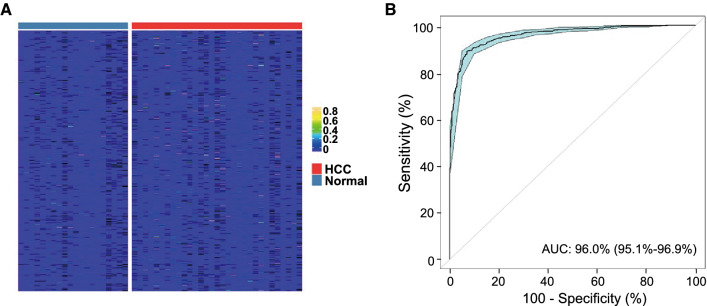
HCC tissue markers were able to classify normal and HCC plasmas. **A** Heatmap of the MHL scores of the 65 MHB tissue classifiers in normal and HCC plasmas samples; **B** normal and HCC plasma samples were classified using the 65 MHB markers by RF method, which demonstrated high degree of accuracy in classification

To construct a cfDNA methylation signature for HCC, we sequenced methylation libraries of cfDNA samples from 35 HCC plasma samples of our study cohort and from 24 healthy individuals. We applied the RF-trained classification model to classify the filtered plasma DNA libraries (Fig. 3A). Results showed that it had an AUC of 96.0% (95% CI 95.1–96.9%) from the ROC curve (Fig. 3B) in identifying HCC plasma.

### DNA methylation markers for the prediction of MVI status

**Fig. 4:**
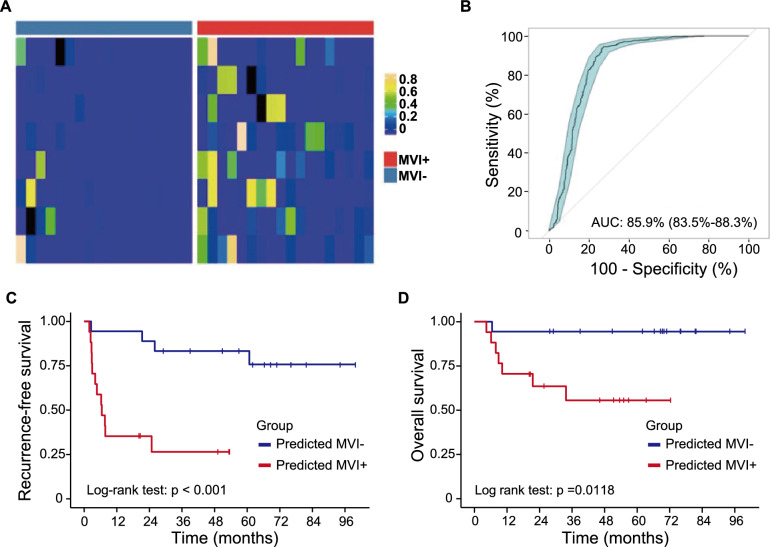
DNA methylation markers differentiate MVI- and MVI + tissues. **A** Heatmap of MHL and UMHL scores of the 8 MVI markers in MVI- and MVI + tissues; **B** RF-built models using discovered MVI markers accurately classified MVI- and MVI + tissues in cross-validation; **C**–**D** recurrence-free survival rate (**C)** and overall survival rate (**D)** of HCC patients in MVI^+^ and MVI^−^ groups predicted by tissue DNA methylation markers. Log-rank test was used

To identify DNA methylation markers that differentiate MVI− and MVI + HCC tissues, we performed Wilcoxon rank-sum test (FDR < 0.05) on the MVI− and MVI + HCC tissues’ DNA methylation profiles on their MHL and UMHL scores, and identified 3 MHL-quantified MHBs and 5 UMHL-quantified MHBs as classifiers for MVI− and MVI + tissues (Fig. 4A). We combined these MHBs’ and trained a Random-forest MVI classification models on our training cohort samples. When being cross-validated, this model showed an AUC of the model was 85.9% (Fig. 4B, 10-time repeats, 95% CI 83.5–88.3%), suggesting that the identified DNA methylation markers consistently and robustly differentiate MVI− and MVI + HCC tissues. Further, survival analysis revealed that RFS rate and OS rate were significantly worse in MVI + group predicted by our methylation markers than MVI- group predicted by our methylation markers, which validated high accuracy of our tissue DNA methylation markers for MVI status prediction (Fig. 4C–D).

### The performance of DNA methylation signature to predict MVI status

**Fig. 5:**
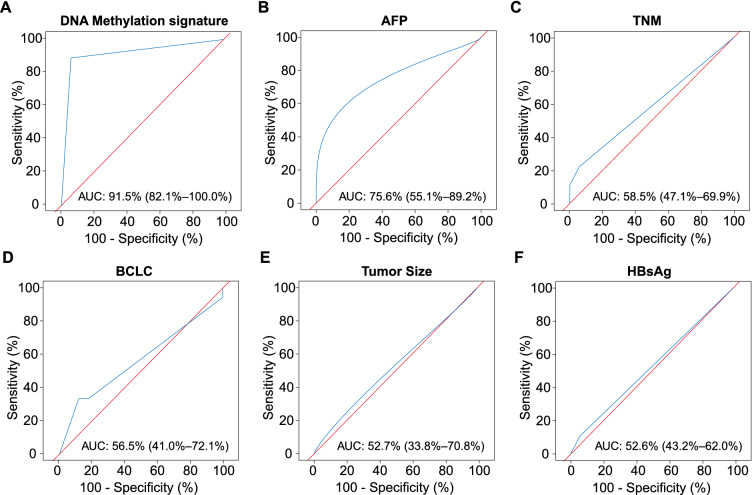
ROC analysis for diagnosing MVI based on the diagnosis results of DNA methylation signature and clinical characteristics. **A** ROC analysis for diagnosing MVI based on the diagnosis results of DNA methylation signature. **B**–**F** ROC analysis for diagnosing MVI based on clinical characteristics included AFP (**B**), TNM (**C**), BCLC (**D**), tumor size (**E**), and HBsAg (**F**)

To compare the prediction performance of the DNA methylation signature to the clinical characteristics, we performed ROC analysis for predicting MVI based on the diagnosis results of DNA methylation signature and clinical characteristics, respectively. We found that the AUROC of the DNA methylation signature was up to 91.5% (95% CI 82.1–100.0%) (Fig. 5A). In contrast, none of the AUROCs of clinical characteristics were more than 80.0%: AFP, 75.6% (95% CI 55.1–89.2%); TNM, 58.5% (95% CI 47.1–69.9%); BCLC, 56.5% (95% CI 41.0–72.1%); Tumor Size, 52.7% (95% CI 33.8–70.8%); and HBsAg, 52.6% (95% CI 43.2–62.0%) (Fig. 5B−F). In addition, we also integrated AFP into our tissue DNA methylation markers to examine whether AFP could improve performance of our tissue methylation makers for MVI status prediction (Additional file 2: Figure S2A). Unfortunately, the integrated methylation markers only achieved an AUC of 86.3%, which is not superior to our tissue DNA methylation markers (Additional file 2: Figure S2B).

Furthermore, we performed a univariable and multivariable logistic regression analysis to assess the associations between MVI and the DNA methylation signature or other clinical characteristics. On univariate analysis, variables associated with MVI were the DNA methylation signature (*p* < 0.001) and AFP ≥ 400 ng/mL (*p* = 0.028) (Table 2). The other parameters were not significantly correlated with MVI. At multivariate analysis, only the DNA methylation signature (*p* < 0.001; odds ratio, 47.51, 95% CI 5.74–393.27) was independent risk factors for MVI (Table 2).

**Table 2:** Univariate and multivariate analysis of risk factors for MVI of HCC

Risk factor	Univariate analysis			Multivariate analysis	
	Odds ratio	*P*-value		Odds ratio	*P*-value
Age (years)	0.97 (0.92, 1.04)	0.418			
Gender	0.31 (0.03, 3.34)	0.336			
HBsAg	2.27 (0.19, 27.58)	0.521			
AFP (≥ 400 ng/mL)	7.11 (1.23, 40.98)	0.028*		3.10 (0.26, 36.86)	0.370
Tumor size (cm)	1.2 (0.29, 5.02)	0.803			
BCLC stage	1.46 (0.32, 6.70)	0.628			
TNM stage	0.58 (0.09, 4.01)	0.584			
DNA methylation signature	60.00 (7.48, 481.57)	< 0.001*		47.51 (5.74, 393.27)	< 0.001*

^*^Data are statistically significant results from logistic regression analysis

*AFP* alpha-fetoprotein, *HBsAg* hepatitis B surface antigen

### DNA methylation signature and RFS

**Fig. 6:**
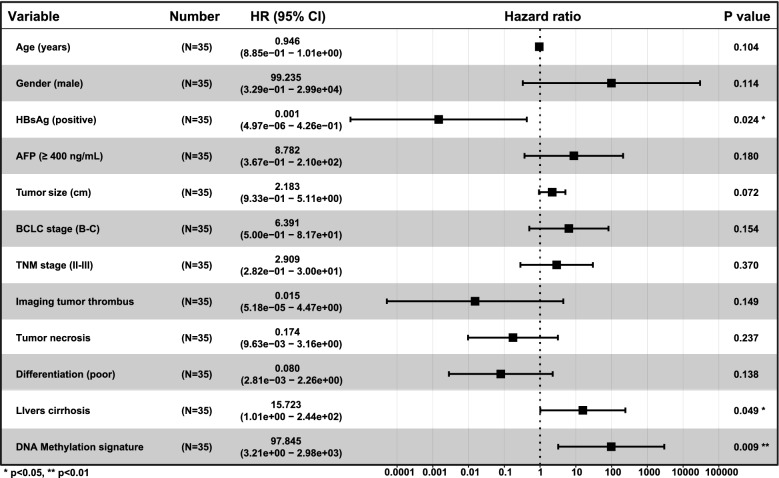
Multivariate Cox analysis of clinicopathologic factors and DNA methylation signature with recurrence-free survival

On univariate analysis, DNA methylation signature was associated with RFS (HR 7.89, 95% CI 2.16–28.88, *p* = 0.002) and by MVI status (HR 32.22, 95% CI 4.06–255.62, *p* = 0.001) (Additional file 5: Table S2). Other factors associated with RFS were AFP, ≥ 400 ng/mL (HR 2.96, 95% CI 1.04–8.39, *p* = 0.042) (Additional file 5: Table S2) and imaging tumor thrombus (HR 4.69, 95% CI 1.44–15.28, *p* = 0.010) (Additional file 5: Table S2). On multivariate analysis, the DNA methylation signature was independently associated with RFS (HR 97.85, 95% CI 3.21–2.98e + 03, *p* = 0.009) (Fig. 6) and by MVI status (HR 8.96e + 02, 95% CI 8.57–9.38e + 04, *p* = 0.004) (Additional file 3: Figure S3).

## Discussion

In this study, we found that methylation pattern could be used as a reliable discriminatory tool for diagnosis of HCC and preoperative prediction of MVI status. First, using genome-wide methylation sequencing through tissues, we identified tissue DNA methylation markers for detection of HCC and MVI. Second, we established a cfDNA methylation signatures, which can be applied to diagnosis of HCC, using patients’ plasma. Overall, our study showed that cfDNA methylation marker could be used for diagnosis of HCC and tissue DNA methylation marker could be applied to preoperative prediction of MVI status.

Noninvasive cfDNA sampling from plasma, that is “liquid diagnosis”, is a promising and reliable method for detection and diagnosis of HCC. A large multi-center study identified a 10-methylation marker panel using cfDNA samples and the panel yielded an AUC of 96.6% in the training dataset and an AUC of 94.4% in the validation dataset for HCC detection, which was superior to AFP [21]. A more recent study identified a 6-methylation marker panel in a phase II clinical study and the model yield a sensitivity of 95.0% and a specificity of 92.0% for HCC detection [22]. Consistent with the results of these studies, we identified a cfDNA methylation signature for HCC detection, which yielded an AUC of more than 95.0%. Our finding together with previous studies, have demonstrated that cfDNA methylation marker have the strong potential for clinical utility in the early detection of HCC.

Preoperative prediction of MVI is of great importance to clinical decisions. Previous studies focused on serum AFP and imaging examination. MVI mainly reflects the biological feature of the tumor, however, we found that despite superiority over other clinical characteristics, the performance of AFP in predicting MVI status is still unsatisfactory, suggesting that AFP cannot fully summarize the biological features of the tumor. Moreover, serum AFP could not predict MVI in AFP-negative HCC. On the other hand, some image features of ultrasound, CT and MRI were predictive of MVI. However, a low accuracy restricted their application. A recent study has identified a 6-gene transcriptome signature associated with MVI based on formalin-fixed paraffin-embedded biopsies and this signature could predict MVI with 74.0% accuracy [28]. Consistent with this study, we developed and established a tissue DNA methylation signature for MVI, which could achieve an AUC of more than 85.0%. In real clinical work, MVI is determined by postoperative pathology examination, but no additional intervention could be done before or during surgery. Routine tumor biopsy could be used for identification of tissue DNA methylation marker and assisting in prediction of MVI status. If clinicians are aware of MVI preoperatively, enlarging surgical margin is a better choice for HCC patients with MVI. Our established tissue DNA methylation signature could realize the goal of preoperative prediction of MVI status, which could assist clinicians in strategy making. Moreover, in addition to predicting MVI status, our DNA methylation signature was prognostic (correlating with RFS), which supports its usefulness for routine biopsy.

This study has several limitations. First, the sample size of this study was small and all samples were from a single center. Second, the performance of preoperative biopsy sample to predict MVI status needs to be further confirmed. Third, preoperative cfDNA signature cannot accurately predict MVI status in this study, although it is very attractive, and subsequent sample size expansion and analytical method optimization may help us develop markers for identification of MVI by liquid biopsy.

In conclusion, we identified and established a cfDNA methylation signature for HCC detection and a tissue DNA methylation signature for prediction of MVI status, which might provide assistance for clinicians in treatment strategies making.

## Supplementary Information

**Additional file 1: Figure S1. **DNA methylation markers classify normal liver and HCC tissues with high accuracy. (A) SVM-built classification models using the 65 MHB markers accurately classified normal liver tissues and HCC tissues, as was demonstrated by the AUC of their ROC curves; (B) Top 10 molecular function categories of the identified MHB markers.

**Additional file 2: Figure S2.** Incorporating serum AFP level into MVI DNA methylation markers did not improve classification accuracy for MVI+ tissues. (A) Heatmap of MHL and UMHL scores of the 8 MVI markers and serum AFP level in MVI- and MVI+ tissues; (B) RF-built models using discovered MVI markers and serum AFP level classified MVI- and MVI+ tissues in cross validation.

**Additional file 3: Figure S3.** Multivariate Cox analysis of clinicopathologic factors with recurrence-free survival.

**Additional file 4: Table S1. **Characteristic information of healthy controls.

**Additional file 5: Table S2. **Univariate Cox analysis of clinicopathologic factors and the DNA methylation signature with RFS.

## Data Availability

The datasets used and analyzed during the current study are available from the corresponding author on reasonable request.

## Abbreviations

HCC: Hepatocellular carcinoma
MVI: Microvascular invasion
TACE: Transcatheter arterial chemoembolization
AFP: Alpha-fetoprotein
ctDNA: Circulating tumor DNA
cfDNA: Circulating free DNA
MHB: Methylation haplotype block
MHL: Methylated haplotype load
RF: Random forest
AUROC: Area under receiver operator characteristics curve
PCA: Principal component analysis
RFS: Recurrence-free survival
OS: Overall survival
