# Supplementary figures and images for: Identification of DNA methylation signatures for hepatocellular carcinoma detection and microvascular invasion prediction
Source: Eur J Med Res. 2022 Dec 5;27:276. doi: 10.1186/s40001-022-00910-w (PMC9720918; doi:10.1186/s40001-022-00910-w)

**A**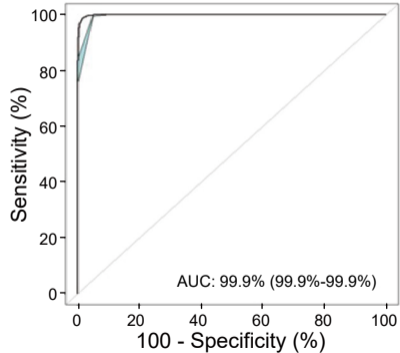**B**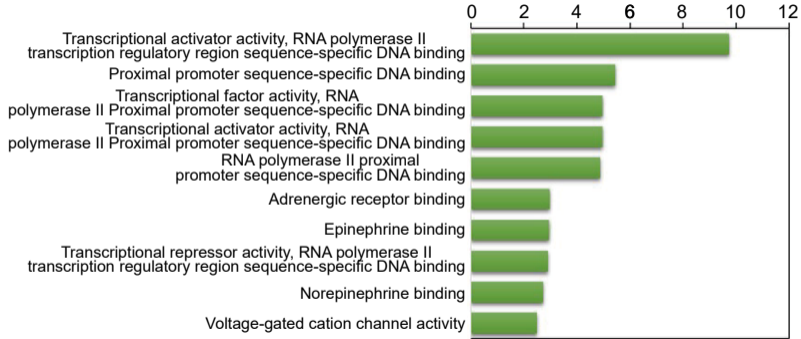

Supplement: Supplementary file 1 — Additional file 1: Figure S1. DNA methylation markers classify normal liver and HCC tissues with high accuracy. (A) SVM-built classification models using the 65 MHB markers accurately classified normal liver tissues and HCC tissues, as was demonstrated by the AUC of their ROC curves; (B) Top 10 molecular function categories of the identified MHB markers. [file 40001_2022_910_MOESM1_ESM.pdf]

**A**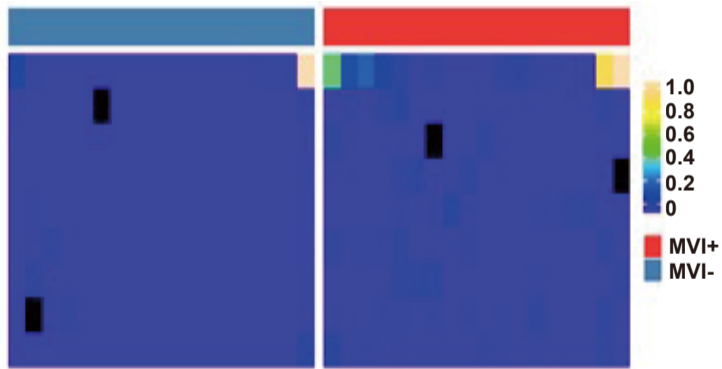**B**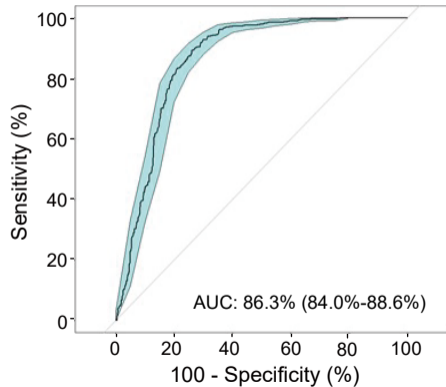

Supplement: Supplementary file 2 — Additional file 2: Figure S2. Incorporating serum AFP level into MVI DNA methylation markers did not improve classification accuracy for MVI+ tissues. (A) Heatmap of MHL and UMHL scores of the 8 MVI markers and serum AFP level in MVI- and MVI+ tissues; (B) RF-built models using discovered MVI markers and serum AFP level classified MVI- and MVI+ tissues in cross validation. [file 40001_2022_910_MOESM2_ESM.pdf]

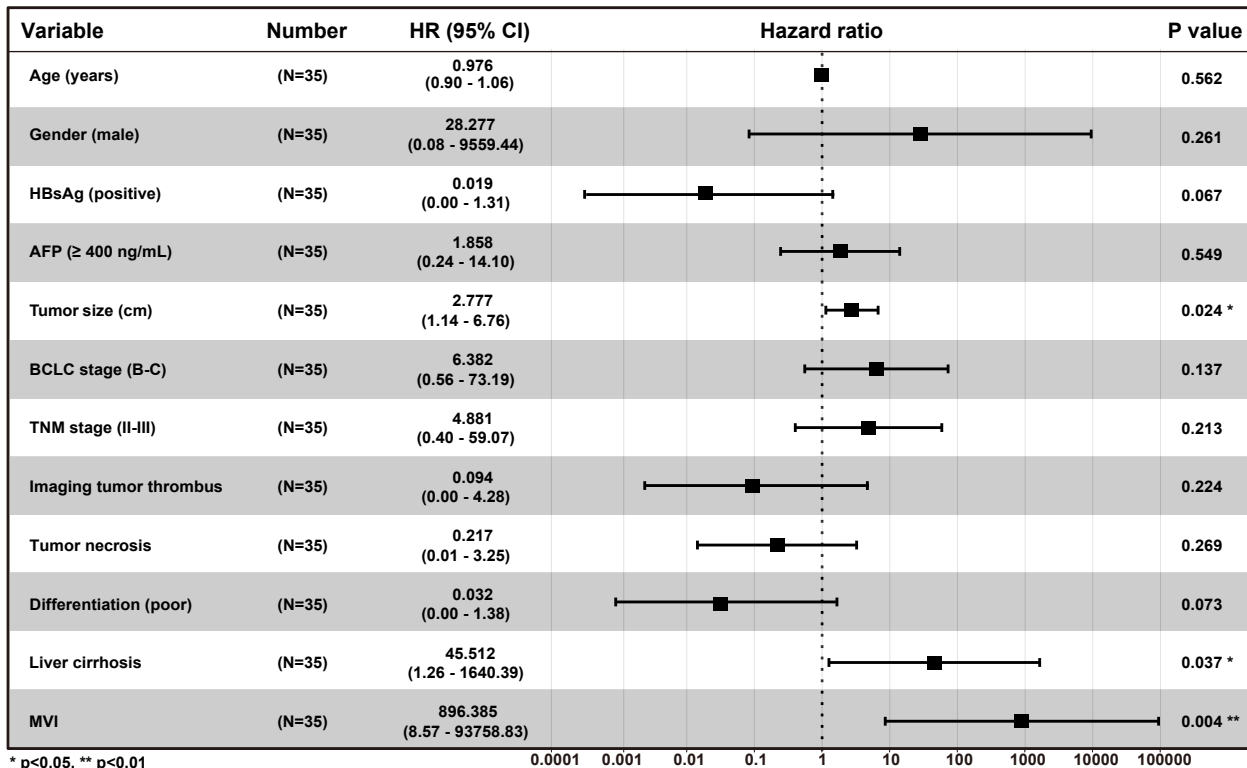

\* p<0.05, \*\* p<0.01

0.0001 0.001 0.01 0.1 1 10 100 1000 10000 100000

Supplement: Supplementary file 3 — Additional file 3: Figure S3. Multivariate Cox analysis of clinicopathologic factors with recurrence-free survival. [file 40001_2022_910_MOESM3_ESM.pdf]
