# Supplementary material for: Identification of DNA methylation signatures for hepatocellular carcinoma detection and microvascular invasion prediction
Source: Eur J Med Res. 2022 Dec 5;27:276. doi: 10.1186/s40001-022-00910-w (PMC9720918; doi:10.1186/s40001-022-00910-w)
Supplement: Supplementary file 4 — Additional file 4: Table S1. Characteristic information of healthy controls. [file 40001_2022_910_MOESM4_ESM.docx]

**Table S1. Characteristic information of healthy controls**

| **Variable** | | **Total (n=24)** |
| --- | --- | --- |
| Age (years) | 55.0 (53.0, 59.0) | |
| Gender |  | |
| Male | 9 (37.5%) | |
| Female | 15 (62.5%) | |
