# Supplementary material for: Identification of DNA methylation signatures for hepatocellular carcinoma detection and microvascular invasion prediction
Source: Eur J Med Res. 2022 Dec 5;27:276. doi: 10.1186/s40001-022-00910-w (PMC9720918; doi:10.1186/s40001-022-00910-w)
Supplement: Supplementary file 5 — Additional file 5: Table S2. Univariate Cox analysis of clinicopathologic factors and the DNA methylation signature with RFS. [file 40001_2022_910_MOESM5_ESM.docx]

**Table S2. Univariate Cox analysis of clinicopathologic factors and the DNA Methylation signature with RFS**

| Risk Factor | Hazard Ratio | P-value |
| --- | --- | --- |
| Age (years) | 0.97 (0.93, 1.02) | 0.238 |
| Gender (female/male) | 2.04 (0.27, 15.55) | 0.491 |
| HBsAg (negative/positive) | 0.59 (0.13, 2.61) | 0.482 |
| AFP (<400/≥400 ng/ml) | 2.96 (1.04, 8.39) | 0.042* |
| Tumor size (cm) | 1.04 (0.79, 1.37) | 0.777 |
| BCLC (0-A/B-C) | 2.51 (0.89, 7.09) | 0.082 |
| TNM (0 -Ⅰ/Ⅱ-Ⅲ) | 2.73 (0.86, 8.67) | 0.088 |
| Imaging tumor thrombus (absence/presence) | 4.69 (1.44, 15.28) | 0.010* |
| Differentiation (well or moderate/poor) | 0.93 (0.21, 4.12) | 0.920 |
| Tumor necrosis (absence/presence) | 2.06 (0.70, 6.06) | 0.191 |
| Liver cirrhosis (absence/presence) | 2.49 (0.70, 8.84) | 0.158 |
| DNA Methylation signature (negative/positive) | 7.89 (2.16, 28.88) | 0.002** |
| MVI (negative/positive) | 32.22 (4.06, 255.62) | 0.001** |

Notes: *p <0.05, **p<0.01

Abbreviations: AFP, alpha-fetoprotein; HBsAg, hepatitis B surface antigen; MVI, microvascular invasion.
